# Acinitis Prostatica1Read at the fortieth annual meeting of the Medical Association of Central New York, held at Rochester, October 15, 1907

**Published:** 1907-12

**Authors:** J. Henry Dowd

**Affiliations:** Genitourinary Surgeon to Mercy Hospital, Buffalo, N. Y.


### Acinitis Prostatica.[1]

BY J. HENRY DOWD, M. D.

Genitourinary Surgeon to Mercy Hospital, Buffalo, N. Y.

### DEFINITION.

AN inflammation of the glandular tissue of the prostate, but especially those situated under the urethrovesicle opening.

To thoroughly grasp the subject under consideration one must be fairly familiar with the anatomy of the prostate not only in health but in disease. Briefly speaking, the prostate is made up of two lobes which are composed of muscles, glandular tissue and their ducts. The middle or third lobe described by the older anatomists is a misnomer and what is found in old men as a tumor between the lobes, and at the neck of the bladder, consists of acinus glandular tissue. In adult life these glands, which are of a racemose variety, situated directly under the urethrovesicle opening and between the lobes, are exactly similar to those found throughout the prostate. In some this glandular tissue is limited in amount; whereas, in others it is very abundant.

These glands together with those of the prostatic lobes, empty their contents into the posterior urethra through eighteen to twenty ducts which are situated laterally and posterior to the

---

1. Read at the fortieth annual meeting of the Medical Association of Central New York, held at Rochester, October 15, 1907

caput gallinaginis. The muscles of the prostate are in two layers, —longitudinal and circular. They surround the glandular substance but not the tubes; in other words, the glandular tubules have no muscular coats. This is an important point to be remembered, showing as it does how any debris from the urethra, when forced into these openings cannot be expelled except by contraction of the prostate at the time of intercourse, or by pressure through the rectum. In health the secretion from these acinous glands commonly called prostatic fluid is thin, of a milky character, alkaline in reaction, containing amyloid, hyaline and lecithin bodies, epithelial cells and amorphous phosphates. If to this fluid is added a 1 per cent. solution of phosphate of ammonia Boetcheis crystals are formed. Prostatic fluid has a double action; first, it increases and dilutes the seminal fluid. Second, it increases the activity of the spermatozoa and acts as a nutrient. One author says in the absence of prostatic fluid the spermatozoa are motionless.

The nervous supply of the prostate and adjacent parts is very marked. It consists of motor, sensory and sympathetic filaments, especially the latter variety. The nerve supply is derived from the inferior hypogastric plexus through the hypogastric plexus which receives its filaments from the aortic plexus, lumbar and first two cervical ganglia. Through these the prostate has connection with practically every sympathetic center in the body. With this intimate relationship it must be very clear why patients with inflammatory disease of this region, should at times suffer such disturbances of the general system, both mental and physical. The close relationship between the prostate, rectum, and anus readily explains disturbances of urination such as retention, strangury, etc., following operations around the sphincter ani muscle.

The vascular supply of this region is very rich, being supplied from the vesicle, hemorrhoidal, and internal pubic. In this connection we can readily account for the frequent involvement, the rapidity with which the prostate enlarges, as in diffuse prostatitis, also the various rectal symptoms, as hemorrhoids, etc., so common in connection with diseases of this organ.

It will only be possible to speak of the cause and infecting agents in the briefest possible manner, and then only of what might be termed the simple variety. The bacteria in question are the gonococci, strepto- and staphylococci and the colon bacilli: the first three being conveyed to the part directly from the urethra and the latter by migration from the rectum. It must not be lost sight of that the disease, gonorrhea, is a mixed infection; there are always two varieties of bacteria present, the gonococci and the strepto- or staphylococci. Authoritative reports are on record where gonococci have been found fifteen years after infection, and in isolated cases I have found them from two to five years afterward. But these are exceptions rather than the rule. In the vast majority of cases gonococci have a limited existence on the same tissue, that is, a membrane may be infected, they develop and flourish for a certain time, producing marked symptoms of inflammation, but under favorable circumstances symptoms subside, discharge becomes less and usually at the end of six to ten weeks no bacteria can be found except the strepto- or staphylococci. These are much in evidence and when they reach inaccessible places, as the acinous glands of the prostate, their existence may depend upon the life of the patient, thus the saying "Once infected with gonorrhea you never recover."

In at least 99 per cent. of the cases of acute acinitis prostatica gonococci are found together with strepto- or staphylococci and occasionally the colon bacilli. The same takes place as in the anterior urethra, when gonococci flourish for a certain time but their existence seems to be limited after the course of a few weeks for, except occasionally, none are found in the expressed secretions. On the other hand, the two other varieties are found as long as the inflammatory action continues.

The infectiousness of these bacteria no one will question. The point I wish to emphasise is: a person having had a gonorrheal involvement of the prostatic glands and recesses and the gonococci having entirely disappeared leaving the strepto- and staphylococci behind, they are just as dangerous as though colonies of the former were present.

Noeggerath says, "Eight hundred out of every one thousand men have had gonorrhea and 90 per cent. of them are never cured."

Fitch says, "Out of every one hundred women marrying men who have had gonorrhea not ten remain well."

These statements were made years ago and at that time were scoffed at, but what a change of opinion investigation and research have wrought; We know that a woman may be infected with pyogeniccocci causing inflammatory involvement of her reproductive organs and that the same may exist for years without any symptoms. We further know that at any time this inflammation may become active resulting in pelvic abscess, salpingitis, oöpheritis or peritonitis followed by a train of symptoms which, sooner or later, drives her to the gynecologist. In this department of medicine we find a sad state of affairs and which are well proven by statistics. 85 per cent. of gynecology is due to infection at the time of sexual contact, and the writer will go on record as stating that fully 70 per cent. of these are due to the strepto- and staphylococci. In from 40 to 50 per cent. of the operations on these organs, 95 out of every 100 ovaries removed, 70 per cent. of sterility and 20 per cent. of blindness is due to the same cause. One step further and I speak as a prophet; venereal diseases will never be stamped out but if the general practitioner who treats most of these cases understood and would explain to his patients the danger to the opposite sex and carry them to complete recovery, gynecology could be dropped as a branch of medicine; untold suffering and thousands of deaths prevented; and, lastly, thousands and thousands would be added to our population annually.

The symptoms vary according to the extent of involvement and seemingly to the bacterial infection present. In many, many cases examined the writer has found that where gonococci were not found in the expressed secretion the symptoms have not been as marked as in those in which they were observed. That involvement of these glands can exist harboring bacteria virulent in nature and no symptoms whatever is undeniable. For that reason I shall try and group a few that will point to the prostate as being the seat of trouble.

First, an acute specific urethritis which after a reasonable time, rational methods having been pursued, the condition refuses to clear up, or the discharge to cease, the urine becomes clear or practically so, when without any provocation suddenly all symptoms with profuse discharge return. Possibly the patient has experienced no symptoms for months, when within twenty-four to thirty-six hours after coitus and free use of liquor a slight gluing of the meatus is noticed. Careful examination may show no gonococci, or possibly only a stray pair on a whole slide, yet in the course of a week or ten days to two weeks a marked urethritis has developed, with no specific cocci to be found.

An epididymitis developing months to years after an acute urethritis and in the absence of all well-known causes as tuberculosis, etc., means an extension from an old inflammatory process in the prostate, especially involving the ejaculatory duct.

Tenesmus, bladder or rectal, varying in degree from only the faintest sensation to frequency of urination increased by slight rigors with an increase of temperature in the absence of, or months after acute infection. Pruritis ani, local conditions being absent. The nervous system suffers markedly, and in neurotic subjects their apparent suffering is most acute. Marked hysteria or neurasthenia in man should cause an examination of the prostatic region. Impotence, either partial or complete, is a common condition.

It is not only atonic but psychical, mostly of the latter variety. On the other hand erections simulating priapism, which in fact might be termed pseudo-priapism are occasionally met. Neuralgic pains of the joints, back and buttock shooting into the extremities, nightly emissions, even in married men, a drop of sticky substance appearing at the meatus following the urine or after movement of the bowels. This substance may not be mucus, spermatic or prostatic fluid, but a symptom telling that the acinous glands of the prostate are full of pus. Painful ejaculation always points to inflammation of this region, the ejaculatory ducts being involved.

Although the treatment is not materially different, differentiation should be made between inflammation of the acinous glands, vesiculitis, follicular prostatitis and involvement of the ejaculatory ducts. It will be admitted without discussion that folliculitis and acinitis prostatica cannot exist as a separate disease, yet one or the other so predominates that the lesser, per se, unless acute, gives no intimation of its presence. The former usually occurs as an acute condition, being accompanied by marked symptoms. The latter usually develops more or less chronically being an extension from the former, or due to local measures used.

Acute folliculitis generally develops during the second or third week of acute urethritis. Nightly calls to urinate range from every half to two hours. Daily calls are increased, and the second glass if examined carefully will be found to contain numerous small particles which are composed entirely of pus, commonly called prostatic plugs. Tenesmus, both bladder and rectal, is at times very marked. Rarely is there any increase of temperature and rigors are unknown, except possibly chilly sensations when urinating, being due to nervous influences.

Neurotic patients may be slightly agitated, but to no marked degree. Digital examination will show no change in the prostate, with the exception of increased heat. If rational methods are used resolutions should be complete, but in the majority of cases the opposite is the case. The symptoms are now similar but of a much lesser degree. Examination per rectum will show the gland to be perfectly normal, and if pressure be made only two or three drops of fluid will appear at the meatus. This to the naked eye will show numerous small particles, prostatic plugs, and frequently ribbon-like substances from the lumen of the follicles, but very little if any free pus. On the other hand, when inflammation has reached the acinous glands already described, and if those in the anterior portion are also involved, symptoms are entirely changed. Here is found a sense of fulness in the region of the rectum, marked pruritis ani, distinct chills and an increase of temperature. Paroxsysmal tenesmus is a prominent symptom, recurrent exacerbations of urethral inflammation and epididymitis with pains in the joints. Rectal examination will show the gland at times markedly enlarged and always one or more nodular swellings at the base of the gland, directly under the urethrovesicle opening. In isolated cases these glands break down and form localised abscesses, which when emptied of their contents leave depressions at times as large as a marble.

Vesiculitis, if acute, can scarcely be mistaken. Urination is increased to from every fifteen to forty-five minutes, temperature runs high and rectal and bladder tenesmus is marked. Digital examination will show the vesicles one or both, enlarged to the size of one's index finger. In the chronic state all symptoms are greatly modified. One that at some time of the day occurs and is almost pathogonomonic, is the escape of a quantity of almost pure pus following urination or while at stool. If found necessary differentiation can be easily accomplished as follows: after having the patient urinate thoroughly cleanse the canal by the aid of an antiseptic irrigation. He should now, with the prepuce retracted, hold the meatus while the operator massages the prostate, avoiding the acinous glands. Any fluid can be caught on a slide and examined at once.

Only prostatic fluid should appear, providing there is no trouble in the follicles. Again wash the urethra and massage the acinous glands avoiding the seminal vesicles, and ampullæ of the ejaculatory ducts. This slide should show no spermatozoa, but pus if they are involved. Again wash the urethra and thoroughly express from the vesicles and ampullæ their contents. Spermatozoa will now appear, and pus if any inflammatory action be present.

*Caution.*---No matter how experienced, the unaided eye cannot be depended upon for a differentiation of fluid appearing at the meatus. Following sexual excitement, and when coitus has not taken place, the writer after massaging the prostate has collected at least one drachm which the microscope showed to be normal prostatic fluid, not containing a single pus cell. Urine which was passed in such cases was quite opaque, but contained no pus.

There is no routine treatment for this condition. Every case must be considered a law unto itself, for what may be beneficial in one may act as a powerful irritant in the next. A few general instructions are very necessary and must be adhered to if any result is expected.

*a.* Total abstinence as to liquor and coitus, even sexual excitement.

*b.* Hygiene and internal medication according as to the urine will show a necessity for.

*c.* Most careful attention by the patient as to treatment, this to be governed by results obtained.

The first and most important step in the case of these patients is a thorough knowledge by both patient and physician of all things that are known to produce irritation. Coitus, even sexual excitement, and liquor are positively interdicted. The diet to a certain extent should be modified, but not to a degree which may cause a loss of tonicity in the tissues.

As a general rule food known to cause increased acidity and concentrated urine should be avoided. Such may be mentioned as shell fish, tomatoes, cucumbers, asparagus, and the like. If the urine shows increased acidity, with an increase in uric acid, red meat should be limited. Above all things local irritation should be avoided. In an occasional case an injection with the hand syringe will be found beneficial, but irrigation, instillations and above all sounds, bougies and electric contrivances are positively contraindicated. The urine is a most valuable aid in treatment and the condition of the nervous system ascertained through this source will, with appropriate medication in the majority of cases, swing the pendulum in the right direction. Thus, if the urine is found alkaline or feebly acid, of a low specific gravity, possibly containing a trace of albumin, iron with nerve tonics are indicated. On the other hand, if the opposite is found sedatives in connection with physical quietude is an absolute necessity. The urine is also the barometer for feeding, for in the first instance a stimulating diet is called for as eggs, etc., whereas, in the latter stimulating food must be avoided to a great extent. The removal of the retained pus is accomplished in two ways; ejaculation during coitus or nightly emissions, and by aid of the finger in the rectum. For the latter the following procedure is advised. Express from the urethra all free pus and the patient should then urinate in two glasses.

The prepuce should now be retracted and the patient told to hold the meatus tightly with the left thumb and forefinger. Having him stoop well over a chair or other support the operator's index finger is introduced into the rectum. With the opposite fingers over the pubes the prostate should be pressed against the examining finger. Pressure is now made and especially in the region of the urethrovesicle opening. In the great majority of cases on one or both sides of the median line these glands can be felt usually, as a slight nodule, occasionally semifluctuating which will become a sort of depression after the contents have been expressed. The finger is now withdrawn and on a glass slide held at the meatus the fluid is collected for examination. Fluid will escape even though the conditions be normal, and the vesicles not touched, but the microscope will decide its nature, whether pus or prostatic fluid. Ten minutes being allowed to elapse he should again pass urine which will be quite opaque and should be submitted to examination. The average interval between treatments is one week, but individual susceptibility may postpone succeeding treatments to ten days or two weeks. This susceptibility can only be discovered by the physician who from appearances and otherwise must decide whether improvement is taking place. After inflammation has once reached these glands no amount of treatment will thoroughly eradicate the pus cells, but it should be continued until only two or three can be found on a field. Treatment should then be suspended when in the course of a month to six weeks if a temperate life has been lead it will be found that nature has eradicated every vestige of the former condition.

In arriving at a cause for this condition and a remedy for its prevention the writer would state; 90 per cent. of the men have or have had gonorrhea and in 100 per cent. the posterior urethra is involved. If in six, eight or ten weeks recovery has not taken place (which does not in 75 per cent. of the cases) by injections and stimulating diuretics, more strenuous methods are used. It is usually the sound and in at least 80 per cent of cases infectious bacteria are forced into inaccessible localities only to be driven therefrom at the time of ejaculation.

*Remedy.* Abolish the sound as a surgical instrument in the treatment of urethritis. Have the most experienced teach the medical student, didactically and clinically, how to rationally treat inflammation of the urethra, and when these cases are beyond infection, instead of how to remove ovaries and tubes.

43 West Tupper St.

